# Characterization of hARD2, a processed hARD1 gene duplicate, encoding a human protein N-α-acetyltransferase

**DOI:** 10.1186/1471-2091-7-13

**Published:** 2006-04-25

**Authors:** Thomas Arnesen, Matthew J Betts, Frédéric Pendino, David A Liberles, Dave Anderson, Jaime Caro, Xianguo Kong, Jan E Varhaug, Johan R Lillehaug

**Affiliations:** 1 Department of Molecular Biology, University of Bergen, N-5020 Bergen, Norway; 2 Department of Surgical Sciences, Haukeland University Hospital, N-5021 Bergen, Norway; 3 Computational Biology Unit, BCCS, University of Bergen, N-5020 Bergen, Norway; 4 Institute of Molecular Biology, University of Oregon, Eugene, OR 97403-1229, USA; 5 Department of Medicine, Thomas Jefferson University, Philadelphia, PA19107, USA

## Abstract

**Background:**

Protein acetylation is increasingly recognized as an important mechanism regulating a variety of cellular functions. Several human protein acetyltransferases have been characterized, most of them catalyzing ε-acetylation of histones and transcription factors. We recently described the human protein acetyltransferase hARD1 (human Arrest Defective 1). hARD1 interacts with NATH (N-Acetyl Transferase Human) forming a complex expressing protein N-terminal α-acetylation activity.

**Results:**

We here describe a human protein, hARD2, with 81 % sequence identity to hARD1. The gene encoding hARD2 most likely originates from a eutherian mammal specific retrotransposition event. *hARD2 *mRNA and protein are expressed in several human cell lines. Immunoprecipitation experiments show that hARD2 protein potentially interacts with NATH, suggesting that hARD2-NATH complexes may be responsible for protein N-α-acetylation in human cells. In NB4 cells undergoing retinoic acid mediated differentiation, the level of endogenous hARD1 and NATH protein decreases while the level of hARD2 protein is stable.

**Conclusion:**

A human protein N-α-acetyltransferase is herein described. ARD2 potentially complements the functions of ARD1, adding more flexibility and complexity to protein N-α-acetylation in human cells as compared to lower organisms which only have one ARD.

## Background

Protein acetylation is a very common modification with a significant impact on several cellular processes. Acetylation occurs both at lysine residues within proteins (N^ε^-acetylation) and at the N-terminus of proteins (N^α^-acetylation). In yeast, N-acetyltransferase 1 (Nat1p) complexes with Arrest defective 1 (Ard1p) to generate a functional NatA protein N^α^-acetyltransferase [1], Ard1p being the catalytic subunit. Proteins with Ser-, Thr-, Gly-, or Ala- N-termini are described to be substrates of NatA after methionine cleavage [2]. The yeast NatB and NatC complexes acetylates different subsets of methionine N-termini [2-4]. Almost all known N-terminally acetylated yeast proteins are products of one of these Nat complexes[5]. Protein N-terminal acetylation is generally believed to be a cotranslational process linked to the ribosome [6-10]. hARD1, the human protein with highest sequence similarity to yeast ARD1, has been described on the genomic (TE2, GenBank [NM_003491]) [11], mRNA [12], protein, and enzyme activity levels [6]. Endogenous hARD1 was demonstrated to interact with NATH and express protein N^α^-acetyltransferase activity. The complex was found to interact with ribosomal subunits supporting its function in cotranslational acetylation [6]. *In vitro *translated mouse homologues, mNAT1 and mARD1, have also been shown to interact and express N-acetyltransferase activity [13]. In *S. cerevisiae *and *D. melanogaster*, a third subunit of the NatA complex has been described and named Nat5p and San, respectively [8,14]. The function of this subunit is unknown, but sequence analysis suggests that Nat5p/San is an acetyltransferase. The human orthologue, hNAT5, was also recently demonstrated to be a part of the human NatA complex [15].

Even though 80–90 % of all mammalian proteins and 50 % of yeast proteins are estimated to be cotranslationally N^α^-acetylated [4,16-20], only a few examples exist describing the functional importance of proper N^α^-acetylation. For instance, the function of the yeast proteins Orc1p and Sir3p in telomeric silencing is dependent on proper NatA-mediated N^α^-acetylation of these proteins [21,22].

Using yeast null strains, NatA activity has been demonstrated to be associated with G_o _entry, cell growth, and the ability to sporulate [23-26]. The importance of protein N^α^-acetylation has also been described in *C. elegans*, where knockdown of either the ard1 or nat1 homologues resulted in embryonal lethality [27]. The human NatA complex has also recently been demonstrated to be essential for normal cellular viability. RNA interference mediated knockdown of NATH or hARD1 induced apoptosis in HeLa cells [28].

Mouse ARD1 was also reported to be implicated in the acetylation of lysine 532 of HIF-1α, contributing to its degradation in normoxia [12]. However, several independent investigations have reported that at least the wildtype hARD1 protein does not mediate N^ε^-acetylation of the lysine residue 532 of HIF-1α [29-32].

The *hARD1 *gene is located on chromosome X (Xq28). Database searches revealed the presence on chromosome 4 (4q21.23) of a putative human paralogue of the previously published *hARD1 *gene (**GeneID:84779**, hypothetical protein [**MGC10646**]). We named this hypothetical human ARD, hARD2.

Here we describe the cloning and expression of hARD2. The entire ORF of hARD2 is intronless, resembling a gene duplicate. Many gene duplicates are non-functional pseudogenes but some, including hARD2, are active genes producing mRNAs and proteins [33-35]. Similar to hARD1, hARD2 interacts with NATH and expresses N-α-acetyltransferase activity.

## Results

### hARD2 cloning and expression

**Figure 1:**
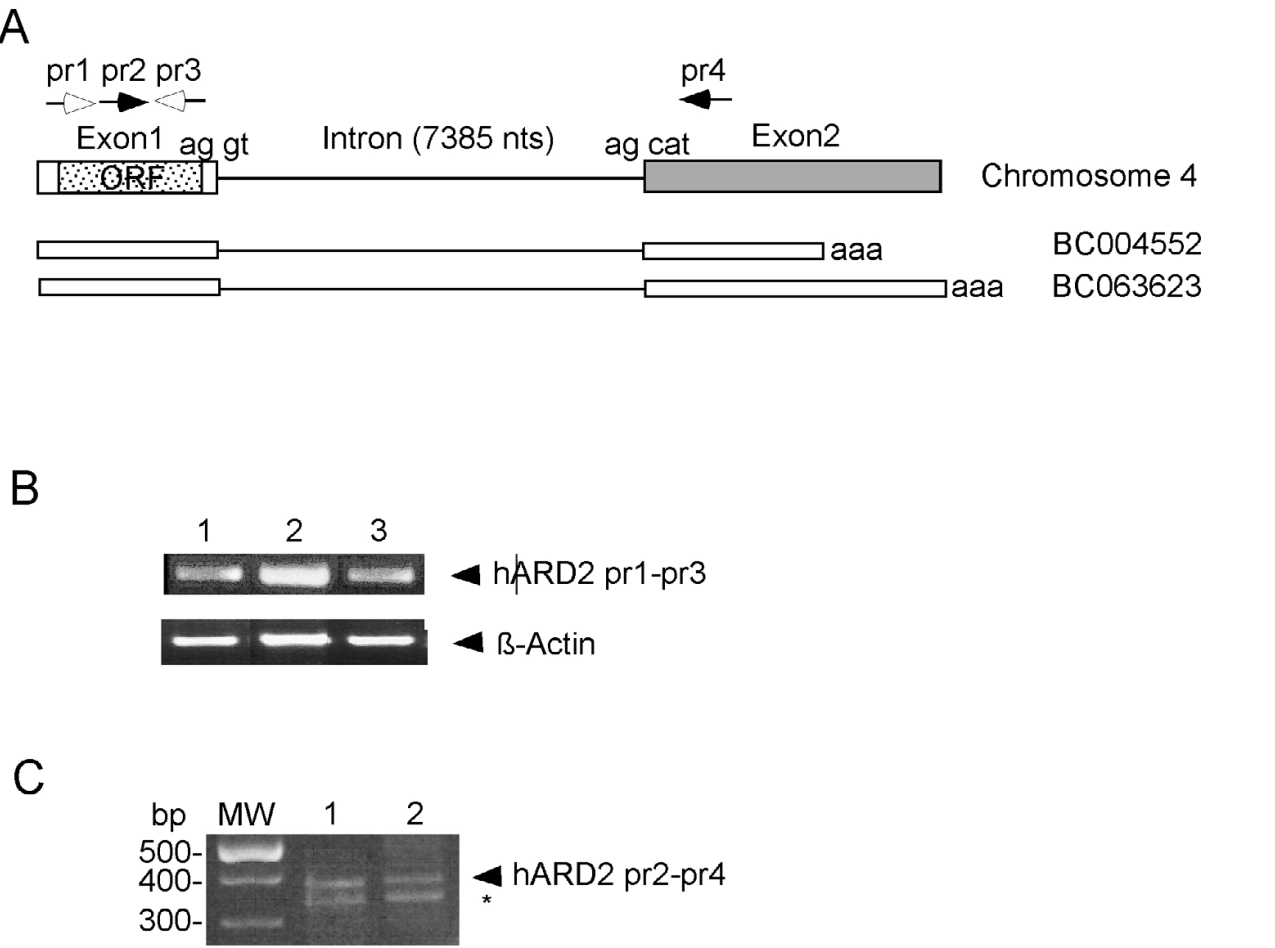
Human ARD2 gene expression. (A) The genomic organization of the hARD2 gene on Chromosome 4 (not to scale) displaying Exon1, Exon2, Intron, the open reading frame (ORF), the nucleotides in the splice sites and the primers pr1-pr4. The registered cDNA sequences BC004552 and BC063623 are also indicated. (B) RT-PCR of the hARD2 ORF using the primers pr1 and pr3 in the cell lines Jurkat (1), HEK293 (2), NPA (3). β-Actin is used as an internal control. (C) Detection of hARD2 exon 1–2 specific PCR product (381 nts) using primers pr2 and pr4 in the cell lines Jurkat (1) and HeLa (2). The asterisk denotes an unspecific PCR product.

Analysis of the genome sequence of *hARD2 *suggests that the complete open reading frame is located within only one exon. This is supported by the sequences of cDNAs [**BC004552**and **BC063623**] (Figure 1A). One intron (7385 nts) and a non-coding second exon was predicted at the 3'-end of the gene. The intron borders are defined by the common "GT-AG" consensus (Figure 1A) [36]. Using RT-PCR, human cDNA and primers covering the predicted ORF, we cloned *hARD2 *(see Methods) and demonstrated *hARD2 *mRNA expression in different cell lines (Figure 1B). To verify the presence of spliced *hARD2 *mRNA we used primers flanking the intron. RT-PCR product of the expected size was detected (Figure 1C) and DNA sequencing verified its authenticity as a spliced *hARD2 *mRNA. There has also been reported an *hARD2 *cDNA sequence covering another exon, 5' to the Exon 1 indicated in Figure 1A [Acc:**XM_496704**]. This cDNA encodes a protein with extra amino acid residues in the N-terminal domain of the protein, while the reading frame is intact. However, we were not able to verify the existence of such a transcript (data not shown).

**Figure 2:**
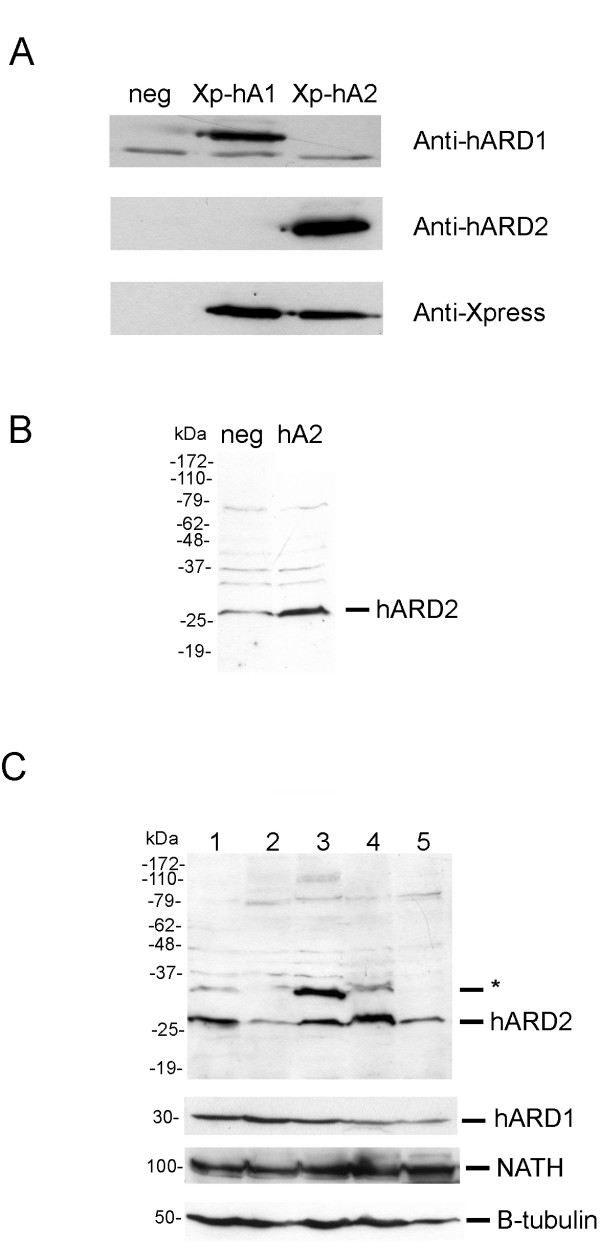
Detection of hARD2 protein. (A) HEK293 cells were transiently transfected with plasmids encoding Xpress-lacZ as a negative control (neg), Xpress hARD1 (Xp-hA1) or Xpress hARD2 (Xp-hA2) and after 48 h processed by SDS-PAGE and Western blotting. Different membranes were incubated with anti-hARD1, anti-hARD2 and anti-Xpress as indicated. (B) HEK293 cells were transiently transfected with a plasmid encoding native hARD2 (hA2) or Xpress-lacZ as a negative control (neg) and after 48 h processed by SDS-PAGE and Western blotting. The membrane was incubated with anti-hARD2. (C) Different cell lines were lysed and approximately 8 μg of total protein was analyzed as above. The membrane was incubated with anti-hARD2, anti-β-tubulin, anti-hARD1 and anti-NATH. 1: SK-MEL2; 2: HEK293; 3: HeLa; 4: MCF-7; 5: NB4. The asterisk denotes an unspecific band or a slower migrating hARD2 variant.

We then demonstrated endogenous hARD2 protein expression using an antibody specific for amino acids 192–206 within hARD2, a region specific to hARD2 as compared to hARD1. To confirm the specificity of the antibody, Xpress-hARD1 and Xpress-hARD2 was expressed in HEK293 cells. The resulting cell lysates were analyzed by SDS-PAGE and Western blotting. Anti-hARD2 only detected X-press-hARD2 and anti-hARD1 only detected X-press-hARD1, while anti-Xpress detected both proteins as expected (Figure 2A). Then, HEK293 cells were transfected with a plasmid encoding wildtype hARD2 and the resulting Western blot displayed a strong band at the expected size of hARD2. In the untransfected control (neg) there was a weaker band at the same position strongly indicating the presence of an endogenous hARD2 (Figure 2B). Endogenous hARD2 protein was detected in several human cell lines indicating that it is commonly expressed in human cell cultures (Figure 2C). Particularly high expression was detected in the MCF-7 breast carcinoma cell line. The expression of hARD1 in the same cell lines indicates that hARD1 and hARD2 are simultaneously present in the cells (Figure 2C).

### Evolution of the hARD2 gene

**Figure 3:**
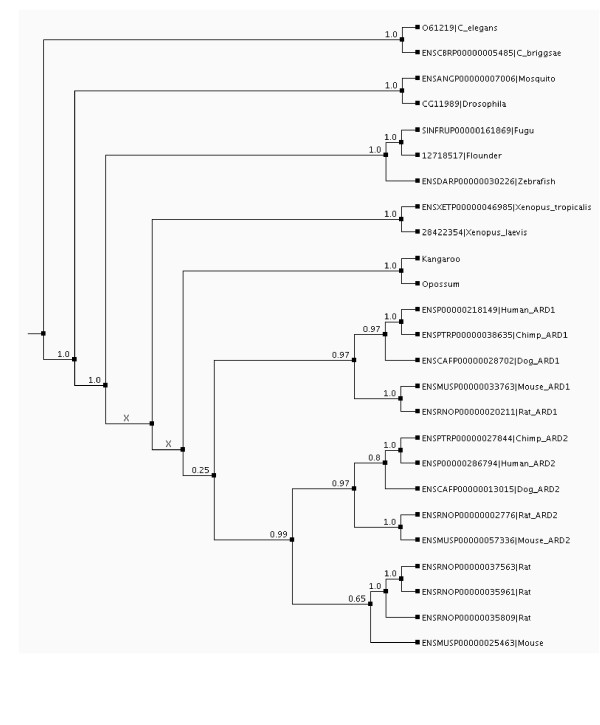
Phylogenetic tree of ARD genes. Phylogenetic tree showing that the speciation resulting in the marsupials, probably precedes the gene duplication that resulted in ARDs. All identifiers are from Ensembl, except for 061219 (UniProt), 12718517 and 28422364 (GenBank GI numbers), Opossum (Ensembl prediction 'Built_from_P41227_and_others_1') and Kangaroo (which is a consensus sequence of all the Kangaroo sequences described in this paper). The protein sequences were aligned using T-Coffee, a coding sequence alignment was produced from this, the best aligned section was extracted (positions 53–214 in the protein alignment, positions 157–642 in the coding sequence alignment), and from this a new tree was generated using MrBayes (3000000 generations, 250000 burn-in, different rates for transitions and transversions, gamma distributed rates across sites). This tree was then rooted by mapping it to the NCBI tree of life whilst miniming the number of gene duplication and loss events when allowing poorly supported branches to be rearranged (Berglund, Steffansson, Betts and Liberles, Manuscript submitted). The figures on the branches are posterior probabilities produced by MrBayes. The two branches marked 'X' are the result of rearrangements during the rooting of the tree.

The hARD1 and hARD2 GenBank identifiers were used to identify the relevant protein family in TAED [37]. The protein sequences of all members of this family were blasted against all Ensembl peptides , identifying them as members of Ensembl family 'N TERMINAL ACETYLTRANSFERASE COMPLEX ARD1 SUBUNIT HOMOLOG EC_2.3.1'. A non-redundant set of the union of the TAED and Ensembl families was produced,-a multiple alignment of the peptide sequences was calculated (data not shown), and an initial phylogenetic tree was produced. Using this tree and the synteny information available through Ensembl suggested that ARD2 is a mammalian specific duplication. To confirm this we searched for expression of the Ensembl rat and mouse ARD2 genes (data not shown). RT-PCR experiments of rat and mouse cDNA using primers flanking each gene confirmed the expression of the mouse gene ENSMUSG00000046000 (ENSMUSP00000057336), which is in a region of synteny with hARD2, and the rat gene ENSRNOG00000023002 (ENSRNOP00000035961) which is not. All the ARD2s have single exon open reading frames, indicating that they might have arisen through a mammalian specific retrotransposition event. Furthermore, we used cDNA and genomic DNA from kangaroo (*Macropus giganteus*) to check for the presence of ARD gene(s) and in an attempt to pinpoint the time at which the duplication occurred. Degenerate primers made from human hARD1 and hARD2 flanking the ORF and one exon were used to amplify and TOPO-TA clone kangaroo ARD from cDNA and genomic DNA, respectively. Blasting hARD1 and hARD2 against the opossum genome predicted peptides available from Ensembl showed only one ARD gene, with a gene structure the same as hARD1. The Kangaroo and Opossum ARDs and the Ensembl putative Dog ARD1 and ARD2 were aligned with the original set of protein sequences [see Additional file 1], a coding sequence alignment was produced from this [see Additional file 2], the best aligned section was extracted (positions 53–214 in the protein alignment, positions 157–642 in the coding sequence alignment) [see Additional file 3], and from this a new tree was generated [see Additional file 4] All kangaroo sequences clustered together in one clade, with the opossum sequence as an outgroup. The tree therefore suggests the presence of only one kangaroo ARD gene (or several that are much more related to each other than are hARD1 and hARD2). This tree was rooted to give the final tree (Figure 3), which shows that the speciation of Kangaroo and Opossum precedes the gene duplication that results in ARD2. Thus, ARD2 seems not to be present in kangaroo and opossum and thus the emergence of ARD2 probably represents a eutherian mammal specific retrotransposition event. Also, kangaroo muscle tissue was lysed and analysed by SDS-PAGE and Western blotting using anti-hARD1 and anti-hARD2 (data not shown). A kangaroo protein of the expected size was observed when using anti-ARD1 but not when using anti-hARD2. The opossum ARD sequence is identical to hARD1 in the region recognized by anti-hARD1. However, the posterior probability supporting the split separating metatherians from the eutherian ARD1/ARD2 divergence is only 0.25. Further, a band of 700 nucleotides was observed by PCR of genomic kangaroo material, although this kangaroo ARD band did cluster as a metatherian sequence, rather than with the ARD2 clade [see Additional file 4]. Altogether, this provides some level of support for a eutherian origin of ARD2. Knowledge of the kangaroo gene structure and full gene sequence will improve the confidence level of this conclusion.

### Structural comparison between hARD1 and hARD2

**Figure 4:**
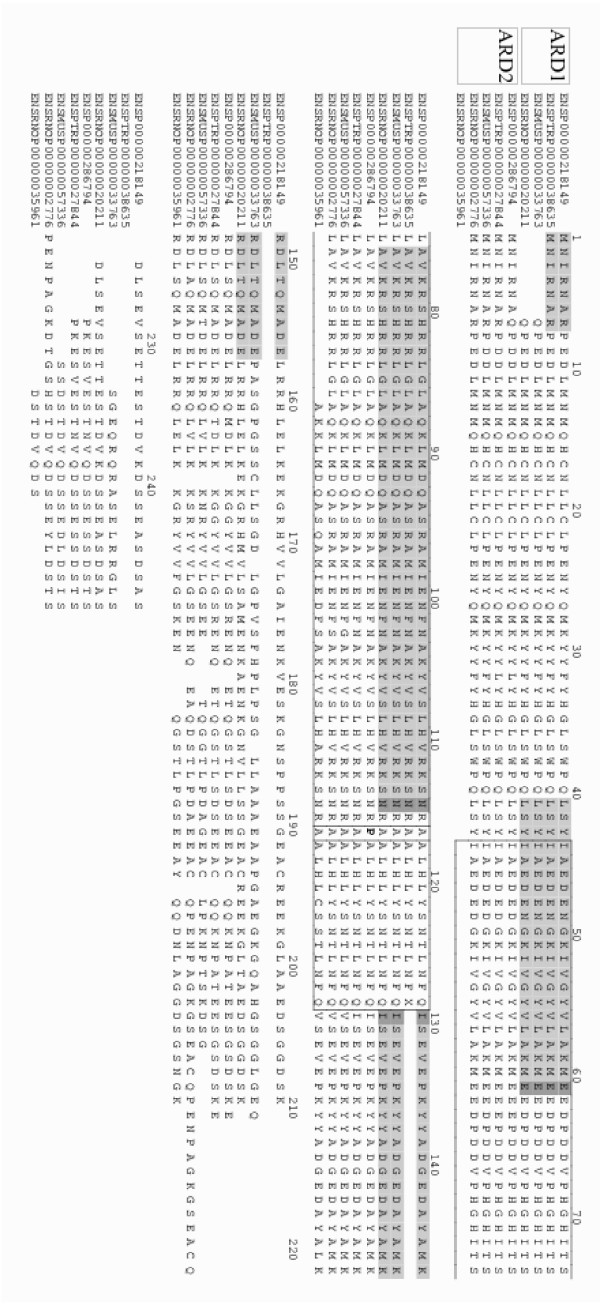
Alignment of selected mammalian ARD proteins. The top four sequences are Human, Chimp, Mouse and Rat ARD1, respectively. The bottom five are Human, Chimp, Mouse and Rat ARD2, plus the additional Rat ARD whose expression was confirmed. ARD1 exons are shown as alternating shaded and unshaded regions and exon boundaries that split a codon are shown in darker shading. The large boxed region is for the acetyltransferase domain (identified by a match to Pfam domain PF00583). The smaller boxed region at position 117 shows the Ala-Pro substituion in human ARD2. Sequence identifiers are from Ensembl.

Alignment of the mammalian ARD1 and ARD2s and identification of the location of their N-acetyltransferase domains (Pfam domain PF00583 [38]), shows only conservative substitutions from ARD1 to ARD2, except for an alanine to proline substitution at aa117 in human ARD2 (Figure 4). As mentioned above, the hARD2 sequence is highly similar to hARD1 in the first ~175 amino acids. The alanine amino acid residue at position 117 within the predicted acetyltransferase domain of hARD1(amino acids 44–129)[39] is substituted by a proline in hARD2. However, structure prediction analysis indicates that this residue is a part of a loop in both hARD1 and hARD2 [see Additional file 5]. This prediction was made by selecting a protein structure that also matches to Pfam domain PF00583 (PDB identifier 1qst, which is for a histone acetyltransferase), aligning it and hARD1 and hARD2 to the Pfam hidden markov model using HMMer [40], running SwissModel [41] and making a Molscript figure [42]. Importantly, this proline residue is not conserved within the mammalian ARD2s (Figure 4). The difference in the C-terminal region may also account for changes in the enzymatic activity. Several serine and threonine amino acid residues have changed between the two proteins and this could result in a change in the regulation of the enzymes by kinases. Prediction of the disorder and globularity [43] of hARD1 and hARD2 indicates that the C-terminal part of these two proteins to some extent diverge (data not shown).

### Overexpressed hARD2 co-immunoprecipitate NATH and HIF-1α

**Figure 5:**
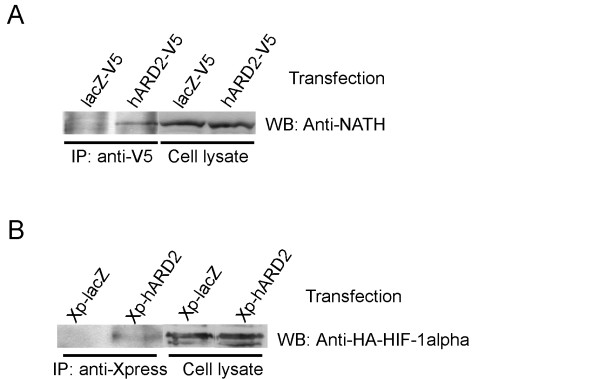
hARD2 interacts with NATH and HIF-1α. (A)HEK293 cells, transfected with plasmids encoding hARD2-V5 or lacZ-V5 as a negative control, were harvested and the lysates were immunoprecipitated (IP) with the anti-V5 antibody. The immunoprecipitates were analysed by SDS-PAGE and Western Blotting. The membrane was incubated with anti-NATH. The amount of lysate loaded on the gel represents approx. 10 % of the material used in the immunoprecipitation reaction. (B) MCF-7 cells were cotransfected with plasmids encoding HA-HIF-1α and X-press-hARD2 or X-press-lacZ as a negative control. After 48 hours the cells were collected and processed as (A). The membrane was incubated with anti-HA.

The NATH-hARD1 complex constitutes a functional protein N-acetyltransferase in human cells [6]. To study whether or not a complex of NATH and hARD2 could exist, we performed immunoprecipitation experiments. Extract of HEK293 cells transfected with a plasmid encoding V5-tagged hARD2 was made and used for immunoprecipitation with anti-V5. Western blotting analysis of the immunoprecipitates revealed that a minor fraction of NATH interacts with hARD2-V5 (Figure 5A). The interaction with NATH is to be expected since the removal of the C-terminal 61 amino acids of hARD1 did not abolish NATH binding [6] and the remaining N-terminal 174 amino acids of the hARD1 sequence is as mentioned above highly similar to hARD2 (Figure 4).

The interaction between hARD1 and HIF-1α has been described [12]. To investigate whether also hARD2 is capable of interacting with HIF-1α, we cotransfected MCF-7 cells with plasmids encoding Xpress-hARD2 and HA-HIF-1α. HA-HIF-1α will then accumulate under these normoxic conditions probably due to saturation of VHL-mediated degradation of HIF-1α. The resulting cell lysates were subjected to immunoprecipitation with the anti-Xpress antibody. Western blotting analysis using anti-HA demonstrated that a fraction of HA-HIF-1α co-immunoprecipitates with Xpress-hARD2 (Figure 5B).

### Subcellular localization of hARD2

**Figure 6:**
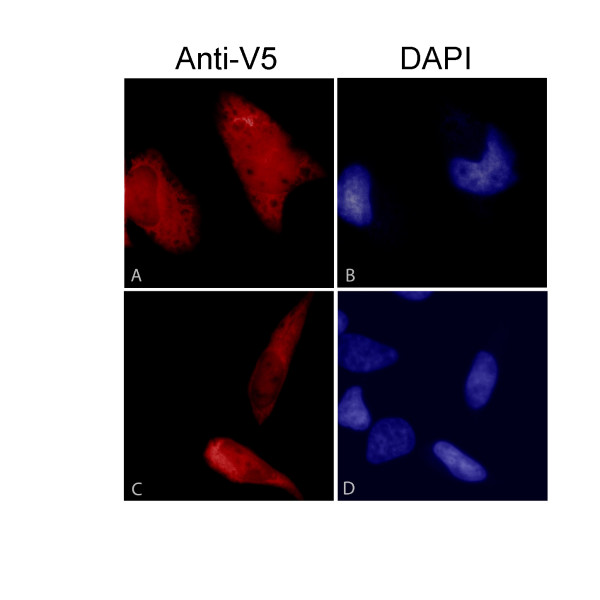
Subcellular localization of hARD2-V5 by immunofluorescence. HeLa cells were transiently transfected with plasmid expressing hARD2-V5, fixed and labelled with anti-V5 and thereafter with Alexa-568-conjugated antibody. Images display hARD2-V5 in red (A and C) and nuclear DAPI staining in blue (B and D). Untransfected cells demonstrating the background staining levels of Alexa-568 can be observed in C next to the transfected cells.

Subcellular localization of hARD2 was studied by expressing V5-tagged hARD2 in HeLa cells followed by immunofluorescence staining (Figure 6A and 6C). Similarly to hARD1 [6], hARD2-V5 was present both in the cytoplasm and in the nucleus, but the majority of the protein appeared to be located in the cytoplasm. Thus, hARD2-NATH complexes may potentially have a function in the cytoplasm.

### N-α-acetyltransferase activity of hARD2

**Figure 7:**
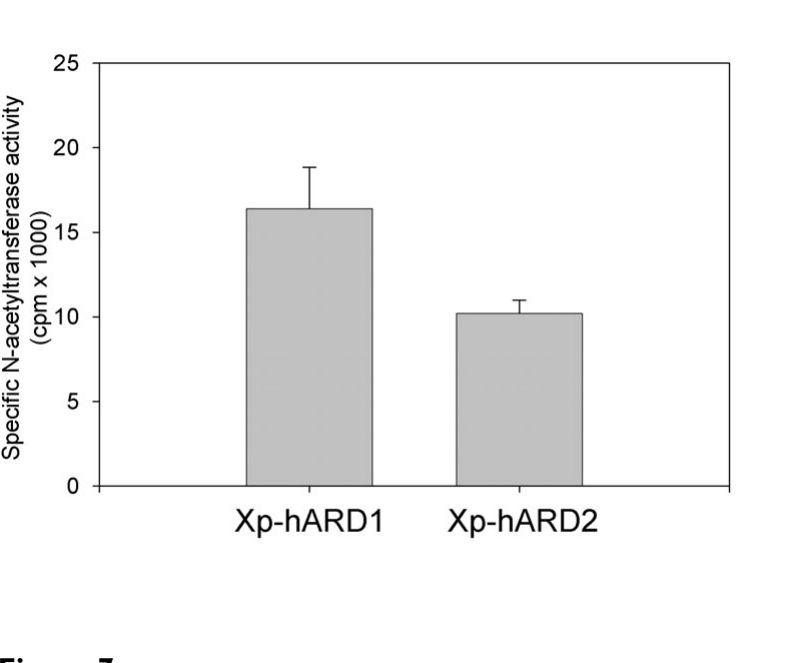
N-Acetyltransferase activity of hARD2. (A) N-terminal acetyltransferase assay using immunoprecipitated Xpress-lacZ (negative control), Xpress-hARD1 or Xpress-hARD2 complexes as the enzyme. Radioactivity [^14^-C] incorporated into the ACTH substrate was determined by scintillation counting. The activity data (cpm) were adjusted according to the FUJIFILM IR-LAS 1000 and Image Gauge v.3.45 relative arbitary units representing levels of Xpress-lacZ/hARD1/hARD2 proteins. The activity of Xpress-lacZ was defined as background and was subtracted from the Xpress-hARD1 and Xpress-hARD2 activity to obtain the specific activity presented.

To investigate whether or not hARD2 is a functionally active protein N-acetyltransferase, Xpress-hARD2 was immunoprecipitated from HEK293 cells and the N-acetyltransferase activity of the Xpress-hARD2 was determined and compared to a negative control using Xpress-lacZ and a positive control using Xpress-hARD1 (Figure 7A). We found that hARD2 expresses N-acetyltransferase activity, demonstrated by the acetylation of the N-terminus of corticotropin (ACTH 1–24). The activity data was normalized using the quantified protein amount in the agarose beads analyzed by Western blotting (data not shown). The radioactivity of the Xpress-lacZ samples were defined as background and subtracted from the Xpress-hARD1/hARD2 values. The activity of the Xpress-hARD2 in three independent experiments gave a mean value of 62 % relative to Xpress-hARD1. These results demonstrate that hARD2 expresses N-acetyltransferase activity, but also suggest that its specific activity is lower than that of hARD1 under the assay conditions used.

### hARD1 and hARD2 are differently regulated during NB4 cell differentiation

**Figure 8:**
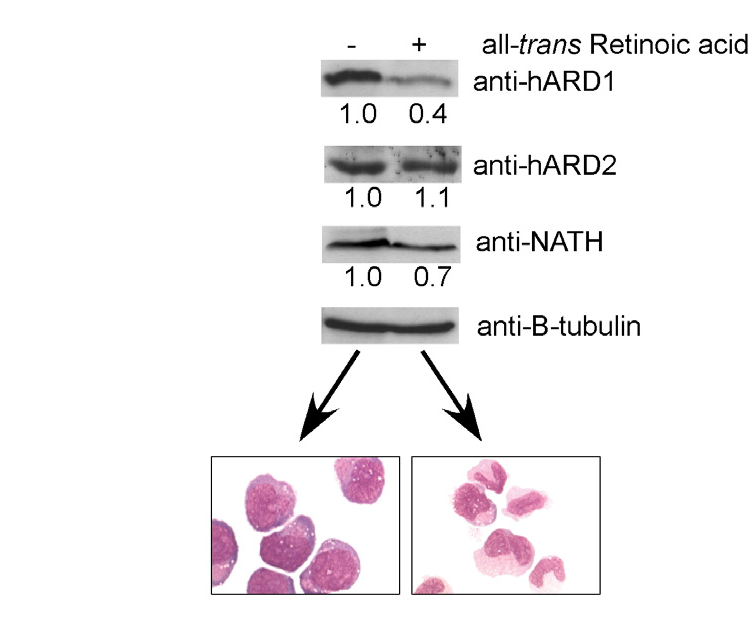
Regulation of hARD1, hARD2 and NATH during differentiation. NB4 cells were treated with 1 μM all-trans retinoic acid for 96 hours. Untreated wells were cultured in parallel as a negative control. Cells were lysed and analyzed by SDS-PAGE and Western blotting. 10 μg total protein was loaded in each well. Membranes were incubated with the indicated antibodies, anti-hARD1, anti-hARD2, anti-NATH and anti-β-tubulin. The data presented was representative of four independent experiments. Protein levels were quantitated using FUJIFILM IR LAS 1000 and Image Gauge 3.45. Protein levels in control (-) samples were set to 1.0 and protein levels in treated cells (+) were estimated relative to this and normalized to B-tubulin levels. Pictures in the lower panel show representative cells after 96 hours of treatment (+) or control (-). Cells were stained using May-Grünwald-Giemsa.

It has been demonstrated that mARD1 and mNAT1 mRNAs are downregulated during neuronal differentiation of P19 cells [13]. Since this suggests a role of these proteins in differentiation, we wanted to investigate the endogenous protein expression of human homologues hARD1, hARD2 and NATH during differentiation. For this purpose we used retinoic acid induced differentiation of the promyelocytic NB4 cell line. Protein levels of hARD1 and NATH significantly decreased during differentiation in NB4 cells (Figure 8). This correlates well with the findings in mouse neuronal cells. However, the levels hARD2 protein is not significantly altered under these conditions (Figure 8).

## Discussion

We here report on the presence of a human gene encoding a protein acetyltransferase at the mRNA, protein and enzyme activity levels. The ARD2 acetyltransferase has originated through a retrotransposition event and represents an additional ARD gene that can evolve independently from ARD1. Our results suggest that the duplication event is eutherian mammal specific. Amino acid sequence differences between hARD1 and hARD2 demonstrate that changes already have taken place, particularly in the C-terminal region of hARD2. As proposed for hARD1, hARD2 could have several different roles in the cell. The interaction with NATH indicates a role in the cytoplasm, possibly cotranslational N-acetylation. It is likely that hARD1 and hARD2 bind the same region within NATH and therefore compete for NATH binding. The difference in activity between hARD1 and hARD2 could suggest that the ACTH peptide used in this assay is not an optimal substrate for hARD2. It is not unlikely that the substrate preference is different between these two enzymes. A putative HIF-1α associated function is supported by the immunoprecipitation results demonstrating the potential interaction between hARD2 and HIF-1α (Figure 5B). Whether or not HIF-1α is a direct substrate for hARD2 mediated acetylation has not been investigated in this study and recent studies question the role of hARD1 in destabilizing HIF-1α [29-32]. Thus, the potential link between HIF-1α and hARD1/hARD2 remains an unresolved issue.

Unfortunately, the multiple sequence alignment outside of the acetyl transferase domain used for phylogenetic studies is not good enough for evolutionary studies involving calculation of substitution rates at synonymous and nonsynonymous sites or other likelihood ratio tests. Future sequencing from additional mammals should improve alignment quality to enable such studies. The ultimate question regarding differences in function between hARD1 and hARD2 is one of selective pressures and neofunctionalization vs. subfunctionalization (see for example, [44,45]), which requires knowledge of the expression patterns and functions of the ancestral state. The metatherian, frog, and fish sequences, which are currently available, can serve as proxies for the ancestral state as they appear not to have duplicate ARD copies. In an attempt to address the function of zebrafish ARD, an antibody to hARD1 was directed against zebrafish tissue, but no binding was detected, probably due to sequence divergence (data not shown). Future work in zebrafish or other non-eutherian mammal species closer to human can address the function of the ancestral state and the relative selective pressures on hARD1 and hARD2.

There are several indications that hARD1 and NATH may be linked to differentiation. As mentioned, mNAT1 and mARD1 mRNAs are downregulated during neuronal differentiation in mouse [13]. In the present study, we show that hARD1 and NATH proteins were both downregulated after induction of differentiation in promyelocytic leukaemia cells. This is the first description of NAT-ARD1 downregulation induced by differentiation in human cells and also the first verification of downregulation at the protein level. Interestingly, the hARD1 gene was one of twelve genes identified to be elevated in dedifferentiated hepatocellular carcinomas [46]. It should also be noted that the yeast ARD1 gene originally was implicated in controlling the switch between the mitotic cell cycle and developmental pathways [25]. Whether or not hARD1 and NATH influences differentiation *per se*, awaits further studies, but our present results add support to a link between the NATH-hARD1 complex and differentiation. The lack of hARD2 downregulation during granulocytic differentiation could suggest that a specific subset of proteins then is acetylated at a stable level. In contrast, the hARD1 specific substrates would be less acetylated during differentiation. This balance between acetylation of different subsets of proteins could have an impact on the differentiation process itself or alternatively facilitate cellular adaptations associated with the process.

## Conclusion

In summary, we have identified and characterized a human protein N-α-acetyltransferase which probably originates from a recent gene duplication event. The hARD2 protein displays similar properties as hARD1 in terms of subcellular localization and potential interactions with NATH and HIF-1α. Further expression and enzymatic studies are required to assess the overall functional contribution of hARD2 in cellular processes.

## Methods

### hARD2 cloning and expression

NCBI BLAST database was employed in the search of human homologues of yeast ARD1. The second best hit after hARD1 was the protein MGC10646, termed hARD2. Primers pr1 and pr3 were designed to clone the hARD2 gene and to detect the presence of hARD2 mRNA. Plasmid encoding V5- and Xpress-tagged hARD2 was constructed from a gene-specific PCR of cDNA made from total RNA isolated from human ARO cells. PCR product was inserted into the TOPO TA vectors pcDNA3.1/V5His and pcDNA 4/HisMax (Invitrogen). Simultaneously a vector encoding wildtype hARD2 was made using a reverse primer including the hARD2 stop codon. cDNA was made as previously described [47]. The primers for amplifying the hARD2 gene (Figure 1) were as follows: pr1 (hARD2 forward), 5'-ATG AAC ATC CGC AAC GCT CAG-3'; pr3 (hARD2 rev), 5'- GGA GGT GGA ATC CGA GCT TTC-3'; pr2 (hARD2 545forward), 5'-CAG CAC ACT TTC TGA TTC TGA AG-3'; pr4 (hARD2 926reverse), 5'-GTA ATG GCA GGT CTC AAA GTTC-3'. β-Actin primers: Actin-F, 5'- GGC ACC ACA CCT TCT ACA 3'; Actin-R, 5'- AGG AAG GCT GGA AGA GTG 3'. Primers for amplifying genomic *M. giganteus *ARD: For: 5'-gtg aar cgy tcn cac cgg cgc cty ggy ctg-3'; Rev: 5'-ctc ttc ctg rcr tgy agr gas acr tay ttk gc-3'. Primers for amplifying *M. giganteus *ARD from cDNA: kARD1/2 ORFf, 5'-ATG AAC ATC CGC AAY GCK MRG CCA GAS GAC C; kARD1/2 ORFr, 5'-CTA GGA GGY KGA RTC SGA GSY YTC TGA GCT GTC C (r = a + g, y = c + t, n = a + c + t + g, s = c + g, k = g + t).

### Cell culture and transfection

Cells were cultured at 37°C, 5% CO_2 _in DMEM (HEK293, embryonal kidney, ATCC CRL-1573 and HeLa, epithelial cervix adenocarcinoma, ATCC CCL-2), RPMI 1640 (NB4, acute promyelocytic leukemia, DSMZ ACC 207 and MCF-7, epithelial mammary gland, breast adenocarcinoma, ATCC HTB-22) or EMEM (SK-MEL2, malignant melanoma, ATCC HTB-68) supplemented with 10% FBS and 3% l-glutamine. Transfections were performed using Fugene6 (Roche) according to the instruction manual. The plasmid pHA-HIF-1α has been described [48]. A hARD2 specific rabbit antibody was generated by Biogenes GmbH using a peptide corresponding to amino acids 192–206 of hARD2. Western blotting was performed as described [47]. Dilutions: anti-hARD2 1:500; anti-hARD1 [6] 1:500; anti-NATH [6] 1:500; anti-V5 (Invitrogen) 1:1000; anti-β-tubulin (Sigma) 1:1000; anti-HA (Sigma) 1:1000.

### Immunofluorescence and immunoprecipitation

HeLa cells were transfected using Fugene6 and grown 24 h on coverslips. Then cells were prepared for immunofluorescence as described [6]. HEK293 or MCF-7 cells (~2 × 10^6^) were transfected using Fugene6 and incubated 48 hours before harvesting and lysis in 300 μl lysis buffer. Immunoprecipitation was performed as previously described [6].

### N-α-acetyltransferase assay

Immunoprecipitation of X-press-lacZ, X-press-hARD1 or X-press-hARD2 was performed as described above. Pellets of Protein A/G-Agarose bound X-press-hARD2 was added 10 μl ACTH (0.5 mM, human corticotropin fragment 1–24, Calbiochem), 4 μl [^3^H]Acetyl-CoA (1 μCi, 107 GBq/mmol, Amersham Biosciences) and 136 μl 0.2 M K_2_HPO_4 _(pH 8.1). The mixture was incubated for 2 hours at 37°C. After centrifugation the supernatant was added to 150 μl SP Sepharose (50% slurry in 0.5 M Acetic acid, Sigma) and incubated on a rotor for 5 min. The mixture was centrifuged and the pellet was washed three times with 0.5 M acetic acid and finally with methanol. Radioactivity in the ACTH containing pellet was determined by scintillation counting.

### Alignment and tree building

Peptide sequence alignments were made using T-Coffee [49] with the default settings. Coding sequence alignments were produced by aligning the coding sequences with reference to the alignment of the corresponding peptide sequences. The phylogenetic tree from the initial peptide sequence alignment was built using MrBayes [50] with the Jones matrix of substitution run on four MCMC chains for 750000 generations, to generate a majority rule consensus tree of all compatible partitions of the final 500000 generations (sampled rate 100). MrBayes was also used to produce the coding sequence alignment based tree, with the same settings as before except for a nucleotide model of substitution allowing different rates for transitions and transversions and a gamma distribution of rates across sites. Gene trees were rooted by mapping them on to the NCBI tree of life whilst minimizing gene duplication and loss events and allowing poorly supported branches (those with posterior probabilities less than 0.7) to be rearranged according to the NCBI taxonomy as a reference species tree (Berglund, Steffansson, Betts and Liberles, Manuscript submitted).

## Abbreviations

ARD, Arrest-defective; DAPI, 4',6-diamidino-2-phenylindole; hARD1, human ARD1; hARD2, human ARD2; HIF-1α, hypoxia inducible factor-1α; mARD1, mouse ARD1; mNAT1, mouse NAT1; NAT, N-acetyltransferase; NATH, NAT human; PCR, polymerase chain reaction.

## Authors' contributions

TA planned the study, participated in all experiments and wrote the manuscript draft. MB and DAL performed the evolutionary analysis and structure prediction. FP performed the differentiation experiments. All authors took part in planning and manuscript preparation.

## Supplementary Material

Additional File 1 Peptide sequence alignment of ARD sequences, produced using T-Coffee. Click here for file

Additional File 2 Nucleotide sequence alignment of ARD sequences produced by aligning the nucleotide sequences as per their peptide sequence alignment given in Additional file 1. Click here for file

Additional File 3 Well aligned region of the alignment in Additional file 2 (positions 157 to 642), with genomic kangaroo ARD fragments added. This alignment was run through MrBayes to produce the unrooted tree in Additional file 4. Click here for file

Additional File 4 Unrooted tree produced by running the alignment in Additional file 3 through MrBayes. This tree was rooted by mapping on to the tree of life to produce the tree in figure 3 (see methods). In figure 3 the kangaroo clade has been summarised as one node called 'Kangaroo', and the the two versions of Mouse ARD1 have been replaced by just the Ensembl version. Figure produced using ATV (Zmasek and Eddy, 2001). Click here for file

Additional File 5 Structure of the acetyltransferase domain of hARD2, as modelled by alignment of both hARD2 and PDB entry 1qst to the alignement of Pfam domain PF00583 and then running through SwissModel. The space-filled residue shows the position of the Ala-Pro substitution on going from hARD1 to hARD2, and that this substitution occurs in a loop. Figure produced using MolScript (Kraulis 1991). Click here for file
