# Supplementary material for: Characterization of hARD2, a processed hARD1 gene duplicate, encoding a human protein N-α-acetyltransferase
Source: BMC Biochem. 2006 Apr 25;7:13. doi: 10.1186/1471-2091-7-13 (PMC1475586; doi:10.1186/1471-2091-7-13)
Supplement: Additional File 4 — Unrooted tree produced by running the alignment in Additional file 3 through MrBayes. This tree was rooted by mapping on to the tree of life to produce the tree in figure 3 (see methods). In figure 3 the kangaroo clade has been summarised as one node called 'Kangaroo', and the the two versions of Mouse ARD1 have been replaced by just the Ensembl version. Figure produced using ATV (Zmasek and Eddy, 2001). [file 1471-2091-7-13-S4.doc]

**S4. Unrooted tree produced by running the alignment in S3 through
MrBayes. This tree was rooted by mapping on to the tree of life to produce
the tree in figure 3 (see methods). In figure 3 the kangaroo clade has
been summarised as one node called 'Kangaroo', and the the two versions of
Mouse ARD1 have been replaced by just the Ensembl version. Figure produced
using ATV (Zmasek and Eddy, 2001).**

**
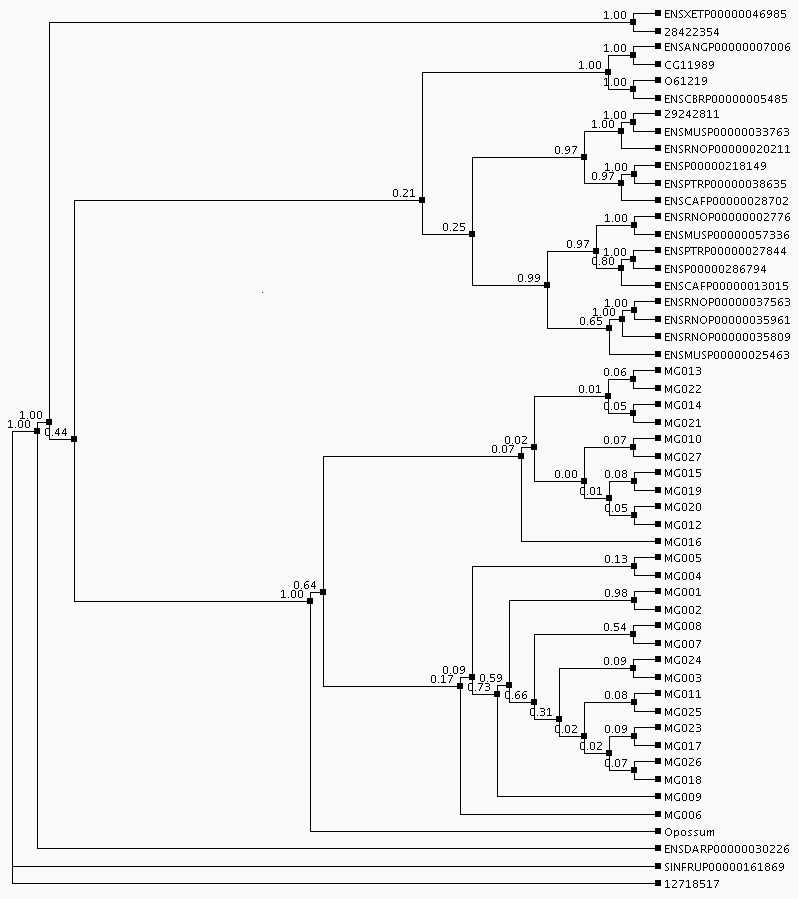
**
