# Supplementary material for: Characterization of hARD2, a processed hARD1 gene duplicate, encoding a human protein N-α-acetyltransferase
Source: BMC Biochem. 2006 Apr 25;7:13. doi: 10.1186/1471-2091-7-13 (PMC1475586; doi:10.1186/1471-2091-7-13)
Supplement: Additional File 5 — Structure of the acetyltransferase domain of hARD2, as modelled by alignment of both hARD2 and PDB entry 1qst to the alignement of Pfam domain PF00583 and then running through SwissModel. The space-filled residue shows the position of the Ala-Pro substitution on going from hARD1 to hARD2, and that this substitution occurs in a loop. Figure produced using MolScript (Kraulis 1991). [file 1471-2091-7-13-S5.doc]

**S5. Structure of the acetyltransferase domain of hARD2, as modelled by
alignment of both hARD2 and PDB entry 1qst to the alignement of Pfam
domain PF00583 and then running through SwissModel. The space-filled
residue shows the position of the Ala-Pro substitution on going from hARD1
to hARD2, and that this substitution occurs in a loop. Figure produced
using MolScript (Kraulis 1991).**

**
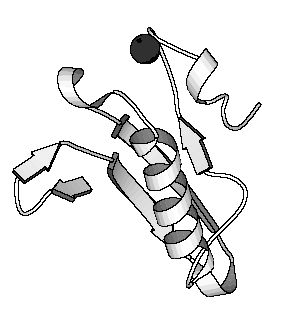
**
